# COVID-19 Information-Seeking, Health Literacy, and Worry and Anxiety During the Early Stage of the Pandemic in Switzerland: A Cross-Sectional Study

**DOI:** 10.3389/ijph.2022.1604717

**Published:** 2022-08-09

**Authors:** Anica Ilic, Katharina Roser, Grit Sommer, Julia Baenziger, Vera Ruth Mitter, Luzius Mader, Daniela Dyntar, Gisela Michel

**Affiliations:** ^1^ Department of Health Sciences and Medicine, University of Lucerne, Lucerne, Switzerland; ^2^ Department of BioMedical Research, University of Bern, Bern, Switzerland; ^3^ Pediatric Endocrinology, Diabetology and Metabolism, Department of Pediatrics, Bern University Hospital, University of Bern, Bern, Switzerland; ^4^ Center for Heart Disease and Mental Health, Heart Institute and Division of Behavioral Medicine and Clinical Psychology, Cincinnati Children’s Hospital and the Department of Pediatrics, University of Cincinnati College of Medicine, Cincinnati, OH, United States; ^5^ Heart Centre for Children, Sydney Children’s Hospitals Network, Sydney, NSW, Australia; ^6^ Centre for Fertility and Health, Norwegian Institute of Public Health (NIPH), Oslo, Norway; ^7^ Department of Gynaecology, Bern University Hospital, University of Bern, Bern, Switzerland; ^8^ Institute of Social and Preventive Medicine, University of Bern, Bern, Switzerland

**Keywords:** COVID-19, information-seeking, media sources, health literacy, worry, anxiety

## Abstract

**Objectives:** To describe COVID-19 information-seeking behavior (CISB) during the first stage of the pandemic in Switzerland and identify its determinants.

**Methods:** We conducted an online cross-sectional survey (4 May to 6 July 2020). Participants self-reported their CISB (information sources and frequency), personal COVID-19 situation (e.g., perception about having had COVID-19), sociodemographic information, and completed validated measures of health literacy, and worry and anxiety. Data were analyzed using descriptive statistics and logistic regressions.

**Results:** We included 1,505 participants (24.7% male; mean age = 43.0 years, SD = 13.9). Most participants reported searching for information daily (*n* = 1,023, 68.0%) and referring to multiple information sources (mean 3.7, SD = 1.5). Commonly used sources were official websites (*n* = 1,129, 75.0%) and newspapers (*n* = 997, 66.2%). Participants with higher health literacy were more likely to seek information daily and use online resources, but less likely to use personal networks than those with lower health literacy. We did not find any association between CISB and worry and anxiety.

**Conclusion:** More opportunities for personal dialogue and education about reliable online information resources should be encouraged to optimize the CISB of groups with lower health literacy.

## Introduction

With the outbreak of the novel coronavirus disease 2019 (COVID-19) extraordinary public health measures have been undertaken and new information was provided almost daily. People were informed about the introduction of diverse restrictive measures and steadily increasing numbers of new cases and deaths. However, at the same time, accurate information about the disease mechanisms and its consequences was lacking [1]. A feeling of uncertainty and fear can result from the fast changes in everyday life, and cause stress [2]. A strategy that can be used to deal with the uncertainty caused by a health threatening situation is searching for information [3–5].

Several definitions of health information-seeking behavior (HISB) have been provided in the literature. In a concept analysis on the term “health information-seeking behavior,” Lambert and Loiselle [4] defined it as the means that individuals use to obtain knowledge about health, including health promotion, risks and illnesses. The authors concluded that HISB involves only active and intentional engagement in information behaviors with the goal of fostering coping and psychosocial adjustment to an illness or health hazard. However, HISB could also result in negative outcomes as disruptive behaviors, information overload or avoidance, distrust, distress and anxiety [4, 5]. Whether information-seeking will result in positive or negative outcomes depends on personal and contextual factors, as well as the fit between the information sought and the retrieved information [4]. A recent study from China found that online COVID-19 information-seeking was associated with higher respect of preventive behaviors against contracting the disease and that higher worry caused by online information-seeking could further enhance preventive behaviors [6]. In a study from the UK, obsessive-compulsive symptoms were linked to higher information-seeking, which in turn resulted in higher adherence to government guidelines on COVID-19 [7]. Another study from Germany, showed two possible and yet contrasting outcomes associated with distress by information [8]. On one side, the experience of distress by information led to better compliance with preventive measures. On the other side, increased distress was also associated with avoidance of information, and in turn with lower compliance to preventive measures. In a study conducted among students in Finland [9], social media exposure resulted in information overload and information anxiety, which occurs when people perceive a gap between what they know and what they think they need to know about a topic [10]. Furthermore, excessive online health information-seeking might lead to cyberchondria, described as excessive anxiety or distress related to a high intensity of online information-seeking [11]. Especially at the beginning of the COVID-19 pandemic, when information was ample and rapidly evolving, it could have caused affective concerns and ultimately led to information avoidance [12]. Information avoidance might bring under-information about the development of the pandemic and its implications on health care and social life, but also affect health-related behaviors and the psychosocial health of individuals.

People’s HISB and its associated outcomes might depend on their health literacy [13]. In a systematic review of health literacy definitions and models, Sørensen et al. [14] define health literacy as one’s ability “to access, understand, appraise, and apply health information in order to make judgments and take decisions in everyday life concerning healthcare, disease prevention and health promotion to maintain or improve quality of life during the life course” (p. 3). The scientific evidence in a wide range of health fields shows that health literacy is associated with health-related knowledge, behaviors and ultimately health care access [15] and health outcomes [16]. In the context of the COVID-19 pandemic, health literacy can affect the approaches used to obtain information on the disease [17]. Inadequate health literacy could result in a poorer understanding of the risks associated with COVID-19, its preventive measures and symptoms, as well as in a higher possibility for misinformation, distrust in health authorities and ultimately adverse mental and physical health outcomes [18]. Previous research assessing the relationship between health literacy and COVID-19 information-seeking behavior focused mainly on university students, digital health literacy and online information-seeking [19–22] or other minority groups as immigrant women [23] and the deaf community [24].

Our aims were to 1) describe COVID-19 information-seeking during the early stage of the pandemic in Switzerland and 2) determine the association between health literacy, worry and anxiety, personal COVID-19 situation, and sociodemographic characteristics and a) frequency of information-seeking; b) use of traditional media; c) use of online resources and d) use of personal networks for information on COVID-19.

## Methods

### Sample and Procedure

The current investigation was part of the larger CoWELL study, a cross-sectional survey that assessed Swiss residents’ psychosocial outcomes and well-being during the early stage of the pandemic. We used a convenience sample. Participants were eligible if they were at least 18 years old, living in Switzerland at the time of the study and provided consent for participation after having received information about the study. Between 4 May and 6 July 2020, we distributed an online survey via Qualtrics software using our personal and professional networks. The survey was available in German, French, and Italian and consisted of two parts. Part 1 included measures of household composition and childcare, personal COVID-19 situation, health-related quality of life, health status, psychological distress, employment situation, health literacy, COVID-19 information-seeking behavior, and sociodemographic characteristics. Participants could also choose to complete part 2, which included assessments of worry and anxiety, post-traumatic growth, post-traumatic stress, resilience, and quality of partner relationship.

### Measurements

#### COVID-19 Information-Seeking Behavior

The current study investigated HISB specific to COVID-19, which will be referred to as COVID-19 information-seeking behavior (CISB). Two questions were developed to assess CISB. First, frequency of CISB was assessed with the following question “How often do you inform yourself about COVID-19?” Answer options included: several times per day, about once a day, several times per week but not daily, about once a week, less than once a week, and no active information-seeking on COVID-19. Second, participants were asked to select the information sources they used to get information on COVID-19. Available options were: TV, radio, daily or weekly newspapers (online or offline), online-only news or portals, official websites, social media, health care providers, family/friends, other sources, and no active information-seeking on COVID-19. Participants indicating other sources were asked to provide further details with an open-ended answer. Multiple answers were possible.

#### Health Literacy

HL was assessed using the 12-item HLS-Q12 [25], a validated short version of the European Health Literacy Survey Questionnaire (HLS-EU-Q; 47 items) [26]. As the HLS-EU-Q, the HLS-Q12 measures HL across three health domains (health care, disease prevention, and health promotion) and four cognitive domains (access, understand, appraise, and apply). For the purpose of the current study, one additional item of the HLS-EU-Q (item 12; judge if the information about illness in the media is reliable) was added to the HLS-Q12 because we considered it important for health literacy associated to COVID-19. Translations of the HLS-EU-Q in the three national languages were provided by the Federal Office of Public Health, and the 13 relevant items were included in the survey. Answers were provided using 4-point Likert scales ranging from “1 = very difficult” to “4 = very easy”. The total health literacy score was then calculated by summing up the scores of the 13 items (possible scoring range: 13–52). The scale (including 13 items) showed very good reliability with a Cronbach’s alpha of 0.89.

#### Worry and Anxiety

Worry and anxiety was assessed using an adaptation of the worry and anxiety questionnaire (WAQ) that can be used to assess worry and anxiety in nonclinical samples [27]. The original WAQ consists of 6 items, including 1 open-ended question (item 1; what subjects do you worry about most often?) and 5 items scored on 9-point Likert scales (scored 0 to 8, with lower scores indicating lower worry and anxiety). Item 5 lists six somatic symptoms (restlessness; fatigue; concentration problems; irritability; muscle tension; and sleep disturbance). Our questionnaire applied 5-point Likert scales, which were recoded into 0 to 8 scoring for the analysis. In compliance with the questionnaire scoring instructions [27, 28], four binary (yes/no) variables and one sum variable were created to assess cognitive symptoms, somatic symptoms, generalized anxiety disorder (GAD), absence of symptoms, and overall worry and anxiety, respectively. To meet cognitive symptoms criteria, participants had to score “4” or higher on items 2, 3, and 4. To meet somatic symptoms criteria, participants had to score “4” or higher in at least 3 of the 6 somatic symptoms of item 5. To meet the GAD criteria, participants had to indicate at least one worry theme in item 1, meet both cognitive and somatic symptoms criteria, and score “4” or higher on item 6 (To what extent does worry or anxiety interfere with your life? For example, your work, social activities, family life, etc.). Participants who did not meet any of the three criteria were attributed “yes” on the fourth variable “absence of symptoms”. Overall worry and anxiety comprised the sum of all Likert-type items (possible scoring range: 0–80). It showed very good reliability with a Cronbach’s alpha of 0.89.

#### Personal COVID-19 Situation

Personal COVID-19 situation included three variables based on self-developed questions and two variables generated from effective public health measures at time of study and from the individuals’ health status relative to the COVID-19 pandemic. We assessed physical distancing behavior with the question “Please select the option that best describes your current situation” (with answer options: isolation; self-isolation; preventive self-isolation; physical distancing; initial physical distancing; no physical distancing), contact with a person who tested positive for COVID-19 with the question “Have you had contact with a positive COVID-19 case?” (yes/confirmed; yes/likely; no), and own perception about having already had COVID-19 with the question “Do you think you have already had COVID-19?” (yes/no). The physical distancing variable levels were recoded into three categories: physical distancing, (self-)isolation (isolation, (preventive) self-isolation), and no physical distancing (initial physical distancing, no physical distancing). We further calculated the stage of the pandemic at study completion according to the date of questionnaire completion: extreme restrictions (up to 10 May, 2020); mild easing of restrictions (from 11 May to 5 June 2020); extensive easing of restrictions (from 6 June 2020). Finally, we defined a binary variable resembling the risk to develop severe COVID-19 (yes/no) based on established risk factors according to the Swiss National Science Task Force [29]. Participants who had a BMI>30 kg/m^2^ (calculated as weight in kilograms divided by height in meters squared) and who reported to have a preexisting chronic condition (including cardiovascular diseases, lung diseases, diabetes, hypertension, a history of cancer) or a transplant were considered at risk for development of severe COVID-19.

#### Socio-Demographic Characteristics

Data on sex (female; male; other), age at study, highest educational achievement (compulsory schooling; vocational training; upper secondary education; university education), and employment situation (employed: full time or part time employment; unemployed: unemployed, on job search, housemaker, retired, invalid or other situation without employment; in education) were collected. Participants indicating “other” sex (*n* = 4) were recoded either as male (*n* = 1) or female (*n* = 3) based on the smallest difference between their height and the average height of men and women in the Swiss population [30].

### Analysis

For participants who completed at least half of items included in the sum variables (overall health literacy and worry and anxiety), we substituted the missing items with the average of the completed items. Missing data in categorical variables were included in the analysis as a separate category.

For aim 1 we used content analysis and descriptive statistics. Open-ended answers to the question on information sources used were coded by the first author (AI), and double-checked by a second author (KR). Disagreement was resolved with discussion. We identified scientific resources, podcasts, employer/colleagues, and unknown internet resources, in addition to the information sources provided in the questionnaire. Sources were grouped into three categories for further analyses: traditional media (TV, radio, newspapers), online resources (online-only news or portals, official websites, social media, scientific resources, podcasts, and unknown internet resources), and personal networks (health care providers, family/friends, and employer/colleagues). The three groups reflect how information is acquired through the sources, with traditional media involving mainly passive information acquisition, online resources assuming mainly active information acquisition, and personal networks implying direct communication and personal interaction.

For aim 2 we first conducted univariable logistic regressions to predict daily information-seeking (yes: several times per day; about once a day/no: several times per week but not daily; about once a week; less than once a week; no active information-seeking on COVID-19), use of traditional media (yes/no), use of online resources (yes/no), and use of personal networks (yes/no) by health literacy, worry and anxiety, personal COVID-19 situation and sociodemographic characteristics. Interactions between health literacy and other independent variables were also included in univariable logistic regression models. Final multivariable logistic regression models included only variables that were significant in univariable regression models. Age, sex, and education were *a priori* included in every multivariable model. We used Wald tests to calculate global *p*-values for all categorical variables included in both univariable and multivariable regression models.

Descriptive statistics and logistic regression analyses were performed using Stata 17 [31] and statistical significance was set at level α< 0.05.

## Results

### Study Population

In total, 1757 persons participated. We included *n* = 1,505 (85.7%) participants who provided complete answers to the items measuring CISB (24.7% male). Of those, 286 (19.0%) did not complete part 2. Participants were on average 43 years old (SD = 13.9). Most participants completed university education (*n* = 957, 63.6%), and were employed (*n* = 1,281, 85.1%). Further information on the sample is displayed in Table 1.

**TABLE 1:** Participants’ characteristics (*n* = 1,505), CoWELL study, Switzerland, 2020.

	Mean	SD	n	%
**Socio-demographic characteristics**				
Sex				
Female			1,129	75.0
Male			372	24.7
Other^a^			4	0.3
Age (years)	43.0	13.9		
18–25			115	7.6
26–40			627	41.7
41–65			655	43.5
66–90			108	7.2
Highest educational achievement^b^				
Compulsory schooling			15	1.0
Vocational training			280	18.6
Upper secondary education			249	16.5
University education			957	63.6
Employment^b^ ^,^ ^c^				
Employed			1,281	85.1
Unemployed			151	10.0
In education			71	4.7
**Personal COVID-19 situation**				
Stage of the pandemic				
Extreme restrictions (up to 10.05.2020)			496	33.0
Mild easing of restrictions (11.05.2020–5.06.2020)			929	61.7
Extensive easing of restrictions (from 06.06.2020)			80	5.3
At risk for severe COVID-19				
No			1,274	84.7
Yes			231	15.4
Physical distancing				
Isolation			2	0.1
Self-isolation			3	0.2
Preventive self-isolation			219	14.6
Physical distancing			982	65.3
Initial physical distancing			277	18.4
No physical distancing			22	1.5
Contact with COVID-19 positive case				
No			1,236	82.1
Yes (confirmed)			154	10.2
Yes (not confirmed)			115	7.6
Perception about having already had COVID-19^b^				
No			1,344	89.3
Yes			156	10.4

a To enable participants indicating “other” sex to be included in the analysis, we classified them as male (*n* = 1) or female (*n* = 3) based on their height [30].

b Variables that had missing data: Education (*n* = 4); Employment (*n* = 2); and Perception about having already had COVID-19 (*n* = 5).

c Participants who indicated multiple employment situations were considered employed if they indicated at least one of the options full-time or part-time employment; in education if at least one of the selected answers was “in education,” but both employment options were not selected; and unemployed if none of the options full-time employment, part-time employment, and education was selected.

COVID-19, coronavirus disease 2019; SD, standard deviation.

The mean overall health literacy score was 42.3 (SD = 6.2; range: 13–52). Further details about health literacy are available in Supplementary Table S1. The mean overall worry and anxiety score was 21.2 (SD = 14.1, range: 0–72). Two thirds of participants did not meet any WAQ criteria (*n* = 733, 67.9%), almost one third met somatic symptoms criteria (*n* = 317, 29.2%), followed by cognitive symptoms criteria (*n* = 118, 10.8%), and GAD criteria (*n* = 56, 5.2%).

### Aim 1: Description of COVID-19 Information-Seeking

**FIGURE 1:**
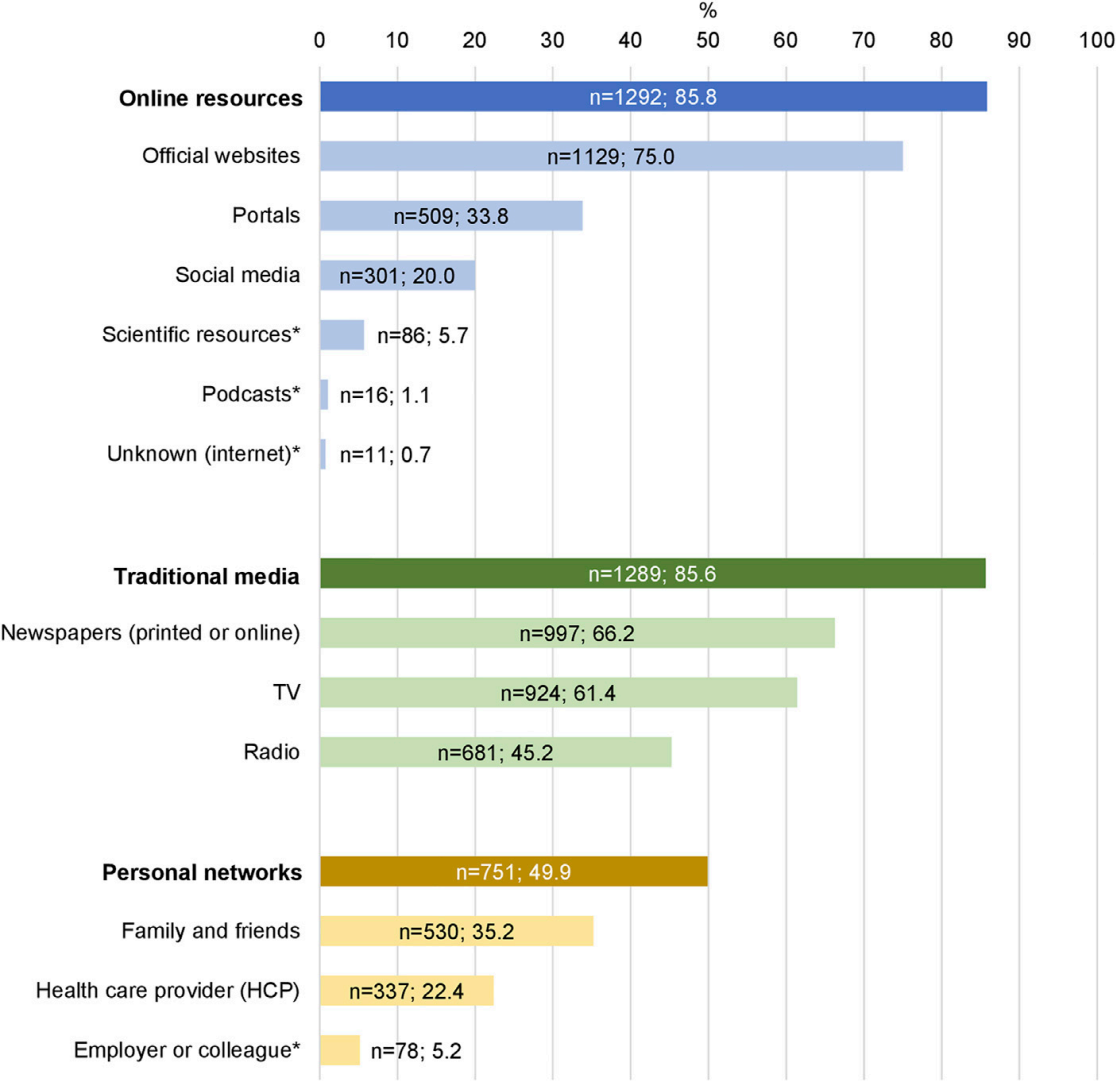
Used information sources (*n* = 1,505), CoWELL study, Switzerland, 2020.* Identified through content analysis of open-ended answers. Note: Totals do not sum up to 100 because of the possibility to provide multiple answers.

Only 1% of participants (*n* = 17) were not actively searching for information on COVID-19. The remaining participants were actively searching for information several times a day (*n* = 377, 25.1%), about once a day (*n* = 646, 42.9%), several times a week but not daily (*n* = 334, 22.2%), about once a week (*n* = 91, 6.1%), or less than once a week (*n* = 40, 2.7%). Participants reported using on average 3.7 different information sources (SD = 1.5). Online resources (*n* = 1,292, 85.8%) and traditional media (*n* = 1,289, 85.6%) were the most used types of sources to obtain information on COVID-19, while only about half of the sample used personal networks (*n* = 751, 49.9%). Often mentioned sources of information were official websites (*n* = 1,129, 75.0%), newspapers (*n* = 997, 66.2%), and TV (*n* = 924, 61.4%). For further details, see Figure 1.

### Aim 2: Associations

The following results are based on separate multivariable logistic regression models for each outcome (Table 2). Detailed results of the univariable regression models are available in Supplementary Table S2.

**TABLE 2:** Results of the multivariable logistic regression models for daily information-seeking, use of traditional media, use of online resources, and use of personal networks, CoWELL study, Switzerland, 2020.

Predictors	Daily information-seeking (*n* = 1,497)				Use of traditional media (*n* = 1,079)				Use of online resources (*n* = 1,495)				Use of personal networks (*n* = 1,497)			
	OR	95% CI		*p*-value	OR	95% CI		*p*-value	OR	95% CI		*p*-value	OR	95% CI		*p*-value
Health literacy																
Overall health literacy score^a^	**1.03**	**1.01**	**1.05**	**0.001**	0.98	0.95	1.01	0.282	**1.03**	**1.00**	**1.05**	**0.028**	**0.97**	**0.95**	**0.98**	**<0.001**
Worry and anxiety																
Overall worry and anxiety score^a^					0.99	0.97	1.01	0.390								
Cognitive symptoms^a^																
Yes (Ref. No)																
Somatic symptoms^a^																
Yes (Ref. No)					0.37	0.08	1.66	0.194								
GAD criteria^a^																
Yes (Ref. No)																
No WAQ criteria^a^																
Yes (Ref. No)					0.39	0.09	1.77	0.225								
Personal COVID-19 situation																
Risk COVID-19																
Yes (Ref. No)	1.14	0.80	1.63	0.475												
Physical distancing^b^				**<0.001**				0.090								
(Self-)isolation	0.80	0.57	1.13		0.76	0.45	1.28									
Physical distancing (Ref.)	**1**				1											
No physical distancing	**0.50**	**0.37**	**0.66**		0.62	0.40	0.96									
Contact with COVID-19 positive case^b^								0.373				0.053				
No (Ref.)					1				1							
Yes (confirmed)					0.68	0.38	1.22		0.96	0.57	1.60					
Yes (probable)					0.78	0.41	1.47		2.84	1.21	6.67					
Thinking to have had COVID-19^a^																
Yes (Ref. No)					0.65	0.38	1.11	0.112					**0.01**	**0.00**	**0.14**	**0.001**
Pandemic stage at study^b^				**<0.001**												
Extreme restrictions (up to 10.05.2020)	**1**															
Mild easing of restrictions (11.05.2020–5.06.2020)	0.79	0.61	1.02													
Extensive easing of restrictions (from 06.06.2020)	**0.32**	**0.19**	**0.55**													
Sociodemographic information																
Sex																
Male (Ref. Female)	**1.39**	**1.04**	**1.86**	**0.024**	**0.60**	**0.39**	**0.92**	**0.019**	0.84	0.59	1.19	0.319	**0.77**	**0.60**	**0.99**	**0.038**
Age^b^				**<0.001**				**<0.001**				**0.049**				**0.001**
18–25 years (Ref.)	**1**				**1**				**1**				**1**			
26–40 years	1.07	0.67	1.70		1.10	0.56	2.16		0.86	0.43	1.72		**0.56**	**0.36**	**0.85**	
41–65 years	**2.43**	**1.50**	**3.94**		**2.62**	**1.28**	**5.34**		0.96	0.48	1.95		**0.54**	**0.35**	**0.82**	
66–90 years	**7.96**	**2.99**	**21.23**		**11.99**	**1.32**	**109.19**		**0.37**	**0.15**	**0.94**		1.03	0.59	1.81	
Highest educational achievement^a^ ^,^ ^b^				0.135				0.801				**<0.001**				0.645
Compulsory school	0.97	0.25	3.84		1.36	0.13	14.08		**0.28**	**0.09**	**0.87**		0.60	0.21	1.73	
Vocational training	0.67	0.49	0.92		0.85	0.50	1.45		**0.38**	**0.26**	**0.56**		0.92	0.69	1.22	
Upper secondary education	0.77	0.55	1.07		1.20	0.68	2.10		**0.57**	**0.38**	**0.86**		1.08	0.81	1.44	
University education (Ref.)	1				1				**1**				1			
Employment^a^ ^,^ ^b^				0.721				0.166				0.414				
Employed (Ref.)	1				1				1							
Unemployed	1.17	0.67	2.04		0.00	0.00	2.89		0.68	0.37	1.23					
In education	1.12	0.63	1.99		0.06	0.00	11.85		0.86	0.39	1.92					
Interactions with health literacy																
Employment * Health literacy^b^								0.119								
Employed (Ref.)					1											
Unemployed					1.18	0.99	1.41									
In education					1.08	0.94	1.22									
Thinking to have already had COVID-19 * Health literacy																
Yes (Ref. No)													**1.11**	**1.05**	**1.18**	**<0.001**

a Variables that had missing data: Overall health literacy score (*n* = 8); Thinking to have already had COVID-19 (*n* = 5); Educational achievement (*n* = 4); Employment (*n* = 2); Overall worry and anxiety score (*n* = 416); Cognitive symptoms (*n* = 410); Somatic symptoms (*n* = 416); GAD criteria (*n* = 425); No WAQ criteria (*n* = 425).

b Global *p*-value from Wald test.

Notes: Statistically significant variables (at level α< 0.05) are highlighted in bold.

CI, confidence interval; GAD, generalized anxiety disorder; OR, odds ratio; WAQ, worry and anxiety questionnaire.

#### Aim 2a: Daily Information-Seeking

We found daily information-seeking to be associated with higher health literacy (odds ratio (OR) = 1.03, 95% confidence interval (CI) 1.01–1.05). The following factors related to personal COVID-19 situation were associated with a lower likelihood of seeking information daily: not following physical distancing measures (compared with respecting physical distancing measures: OR = 0.50, 95% CI 0.37–0.66) and answering the survey during the phase of the pandemic when restrictions were extensively eased since their introduction (compared with the phase with extreme restrictions: OR = 0.32, 95% CI 0.19–0.55). Being male (OR = 1.39, 95% CI 1.04–1.86) and older age (41–65 years: OR = 2.43, 95% CI 1.50–3.94 and 66–90 years: OR = 7.96, 95% CI 2.99–21.23 compared to 18–25 years) were the only sociodemographic variables that were associated with daily information-seeking. Worry and anxiety was not associated with daily information-seeking.

#### Aim 2b: Use of Traditional Media

Older people were more likely than younger people (41–65 years: OR = 2.62, 95% CI 1.28–5.34 and 66–90 years: OR = 11.99, 95% CI 1.32–109.19; reference: 18–25 years) and men were less likely than women (OR = 0.60, 95% CI 0.39–0.92) to use traditional media. Health literacy, worry and anxiety, and personal COVID-19 situation were not associated with the use of traditional media.

#### Aim 2c: Use of Online Resources

Participants with higher health literacy (OR = 1.03, 95% CI 1.00–1.05) were more likely to use online resources. Participants with the following sociodemographic characteristics were less likely to use online resources: those having completed compulsory schooling (OR = 0.28, 95% CI 0.09–0.87), vocational training (OR = 0.38, 95% CI 0.26–0.56), or upper secondary education (OR = 0.57, 95% CI 0.38–0.86; reference: completed university education), and older participants (66–90 years: OR = 0.37, 95% CI 0.15–0.94; reference: 18–25 years). Worry and anxiety and personal COVID-19 situation were not associated with the use of online resources.

#### Aim 2d: Use of Personal Networks

Participants who believed that they had already had COVID-19 (OR = 0.01, 95% CI 0.00–0.14), young and middle-aged adults (26–40 years: OR = 0.56, 95% CI 0.36–0.85 and 41–65 years: OR = 0.54, 95% CI 0.35–0.82; reference 18–25 years), men (OR = 0.77; 95% CI 0.60–0.99) and participants with high health literacy (OR = 0.97, 95% CI 0.95–0.98) were less likely to use personal networks. We further found the interaction between thinking to have already had COVID-19 and higher health literacy (OR = 1.11, 95% CI 1.05–1.18) to be associated with the use of personal networks. We did not find any association between worry and anxiety and use of personal networks.

## Discussion

Respondents reported searching for information on COVID-19 very frequently and using many different sources to get information. We found higher health literacy to be associated with daily information-seeking, use of online resources and less use of personal networks to obtain information on COVID-19. Worries and anxiety did not influence CISB in our sample. Zimmerman [17] showed that during the pandemic people were more likely to use news for health information and to use a higher number of information sources, compared to the period preceding the pandemic. Several sources that report news were often used for CISB in our sample, including newspapers, TV, radio and portals. These sources are common channels of health information for people with lower health literacy [32]. However, our study showed that during a pandemic the use of news sources for obtaining health information is common also among people with higher health literacy. Nonetheless, participants with high health literacy also used online resources to get information; these included mainly official websites that provide public and quality news. Participants with lower health literacy referred more commonly to personal networks. This group might prefer to obtaining health information through dialogue instead of reading, which requires additional comprehension skills. Additionally, it might be more difficult for people with lower health literacy to browse online health information and select reliable information to read, and they might instead prefer being provided with written information [33]. This group might not be aware of the availability of easy-to-access (both in terms of contents and delivery method) and high-quality online information resources. We therefore recommend that efforts aiming at providing information on COVID-19 to people with lower health literacy should concentrate on personal discussion or education about optimal internet navigation and reliable online resources.

An association between health anxiety and information-seeking has been often found in previous research, but there is no evidence that the relationship between the two variables is one-directional [34]. The current study did not find any association between frequency of CISB or any group of information sources and worry and anxiety. A study on the effect of the COVID-19 pandemic on mental health in the Swiss general population identified impaired mental well-being among one third of respondents [35], which is similar to around one third reporting worries and anxiety in our study. A recent study about cyberchondria in the course of the COVID-19 pandemic [36] identified intensive information-seeking as one of the risk factors for greater fear and anxiety. Despite the frequent information-seeking in our participants, we did not identify an association between use of online resources and worry and anxiety. In their concept analysis, Lambert and Loiselle [4] identified two main dimensions of HISB: the information dimension, and the method dimension. The information dimension describes the type (contents) and amount (level of detail) of the search. The method dimension refers to the actions (e.g., reading) performed in order to obtain the information and the used sources (e.g., newspaper). Since our study emphasized on the method dimension, we suggest future research to address the association between worry or anxiety and information type and amount. It is further possible that whether CISB causes further worry and anxiety depends on other aspects than health literacy.

Some aspects related to personal COVID-19 situation were associated with CISB. We found respondents answering during the stage when restrictions were extensively eased to be less likely to seek for information daily. With time people might have gradually decreased the compulsiveness of their searches as a consequence of adaptation to the “new normal.” Additionally, they may have optimized their ideal information path instead of simply avoiding information on the pandemic. However, we have no data on the degree of search detail and time spent to obtain the information. In our study, participants who did not adhere to physical distancing measures did not seek information on COVID-19 daily. In this group the perceived susceptibility to the virus may have been low [37] and resulted in less interest in this topic. Persons who believed to have already had COVID-19 tended not to use personal networks, which might indicate stigmatization of persons with COVID-19 at the early stage of the pandemic [38]. However, among this group, those with high health literacy used personal networks more often than those with low health literacy, indicating that people with a high health literacy might be more capable to communicate about their disease experience with others.

In line with previous literature [39], sex, age and education were associated with CISB. Men were more likely to engage in CISB daily than women, but women used traditional media sources and personal networks more than men. Older participants were more likely to seek for information daily, and preferred traditional media to online resources compared to younger participants. Personal networks were equally important for information-seeking in both old and very young participants, but people in their working age were less likely to use them for information on COVID-19. Older adults might feel higher susceptibility to severe COVID-19. Therefore, they might want to search for information frequently to stay informed about the latest developments. Traditional media might be preferred by this group over online resources because of the ease with which reliable information can be retrieved [33] or familiarity with acquiring health information through these channels [40]. Finally, in line with previous research [39], participants who completed university education reverted to online resources more often than other participants. People with higher education could be more aware of the heterogeneity of online information and trust in their ability to critically assess the retrieved information [41].

### Strengths and Limitations

Our findings should be interpreted in light of some limitations. First, data on the exact contents of the sought COVID-19 information including its quality was not available and our results can only refer to the method dimension of CISB. Second, our findings may be generalized only partially because of study participants’ characteristics. The study used a convenience sample, which included mainly female participants, who were well-educated and highly involved in COVID-19 information-seeking. Our recruitment strategy underlies self-selection bias, with people engaged and interested in COVID-19 being more willing to take a survey on this topic. When data collection started, the state of extraordinary situation was still in place in Switzerland. Therefore, alternative ways to recruit participants were hardly attainable. Due to the characteristics of the sample, the results of the current study might have underestimated the association between worry and anxiety and the assessed aspects of CISB. Therefore, we suggest future research to further explore CISB in populations with lower education and health literacy. The main strength of the current study is its large sample size, which gives more reliable results, better precision, and power. Further, at the moment of data collection, a health literacy assessment tool specific to COVID-19 was not yet available. We used items from the HLS-EU-Q, a well-established health literacy assessment scale which has previously been used in Switzerland and showed very good reliability in our sample.

### Conclusion

Despite the concern that online CISB could further increase worries and uncertainty in people with lower health literacy, our results indicated that this group prefers obtaining information through personal networks. Individuals with lower health literacy should be addressed by using personal dialogue and education about reliable online information resources should be encouraged.

## Abbreviations

BMI, body mass index; CI, confidence interval; CISB, COVID-19 information-seeking behavior; COVID-19, coronavirus disease 2019; GAD, generalized anxiety disorder; HISB, health information-seeking behavior; OR, odds ratio; SD, standard deviation; SNSF, Swiss National Science Foundation; UK, United Kingdom.
