# Supplementary material for: COVID-19 Information-Seeking, Health Literacy, and Worry and Anxiety During the Early Stage of the Pandemic in Switzerland: A Cross-Sectional Study
Source: Int J Public Health. 2022 Aug 9;67:1604717. doi: 10.3389/ijph.2022.1604717 (PMC9395600; doi:10.3389/ijph.2022.1604717)
Supplement: Supplementary file 1 [file Table1.docx]

**COVID-19 information-seeking, health literacy, and worry and anxiety during the early stage of the pandemic in Switzerland: a cross-sectional study**

Supplementary Table S1. Distribution of responses to each health literacy item (n=1505), CoWELL study, Switzerland, 2020.

| Item no.^†^ | Label | Very difficult | Difficult | Easy | Very easy | Substituted | Missing after substitution |
| --- | --- | --- | --- | --- | --- | --- | --- |
|  | On a scale from very difficult to very easy, how easy would you say it is to: |  |  |  |  |  |  |
| 1 (2) | find information on treatments of illnesses that concern you? ^a, 1^ | 10 (0.7%) | 114 (7.6%) | 744 (49.4%) | 622 (41.3%) | 10 (0.7%) | 5 (0.3%) |
| 2 (7) | understand what to do in a medical emergency? ^a, 2^ | 7 (0.5%) | 134 (8.9%) | 736 (48.9%) | 620 (41.2%) | 3 (0.2%) | 5 (0.3%) |
| 3 (10) | judge the advantages and disadvantages of different treatment options? ^a, 3^ | 41 (2.7%) | 440 (29.2%) | 684 (45.4%) | 331 (22.0%) | 3 (0.2%) | 6 (0.4%) |
| 4 (12) | judge if the information about illness in the media is reliable? ^a, 3^ | 81 (5.4%) | 477 (31.7%) | 601 (39.9%) | 334 (22.2%) | 4 (0.3%) | 8 (0.5%) |
| 5 (14) | follow the instructions on medication? ^a, 4^ | 13 (0.9%) | 67 (4.5%) | 428 (28.4%) | 986 (65.5%) | 6 (0.4%) | 5 (0.3%) |
| 6 (18) | find information on how to manage mental health problems like stress or depression? ^b, 1^ | 17 (1.1%) | 180 (12.0%) | 705 (46.8%) | 577 (38.3%) | 20 (1.3%) | 6 (0.4%) |
| 7 (23) | understand why you need health screenings? ^b, 2^ | 10 (0.7%) | 71 (4.7%) | 494 (32.8%) | 918 (61.0%) | 4 (0.3%) | 8 (0.5%) |
| 8 (28) | judge if the information on health risks in the media is reliable? ^b, 3^ | 61 (4.1%) | 408 (27.1%) | 642 (42.7%) | 385 (25.6%) | 2 (0.1%) | 7 (0.5%) |
| 9 (30) | decide how you can protect yourself from illness based on advice from family and friends? ^b, 4^ | 55 (3.7%) | 355 (23.6%) | 642 (42.7%) | 452 (30.0%) | 13 (0.9%) | 8 (0.5%) |
| 10 (32) | find information on healthy activities, such as exercise, healthy food and nutrition? ^c, 1^ | 8 (0.5%) | 50 (3.3%) | 507 (33.7%) | 931 (61.9%) | 1 (0.1%) | 8 (0.5%) |
| 11 (38) | understand information on food packaging? ^c, 2^ | 32 (2.1%) | 239 (15.9%) | 570 (37.9%) | 656 (43.6%) | 1 (0.1%) | 7 (0.5%) |
| 12 (43) | judge which everyday behavior is related to your health? ^c, 3^ | 7 (0.5%) | 76 (5.0%) | 529 (35.1%) | 886 (58.9%) | 0 (0.0%) | 7 (0.5%) |
| 13 (44) | make decisions to improve your health? ^c, 4^ | 22 (1.5%) | 196 (13.0%) | 666 (44.3%) | 611 (40.6%) | 2 (0.1%) | 8 (0.5%) |

^†^ The number in brackets refers to the item number in the original HLS-EU-Q (47-items) questionnaire.

Note: For participants who completed at least half of the items (n=7) but not all 13 items, values of each missing item were substituted with the average score of the completed items. Domains: ^a^ healthcare; ^b^ disease prevention; ^c^ health promotion; ^1^ access information; ^2^ understand information; ^3^ appraise information; ^4^ apply information. Percentages might not sum up to 100, because of rounding.

Supplementary Table S2. Results of the univariable logistic regression models for daily information-seeking, use of traditional media, use of online resources, and use of personal networks, CoWELL study, Switzerland, 2020.

|  | Daily information-seeking | | | | Use of traditional media | | | | Use of online resources | | | | Use of personal networks | | | |
| --- | --- | --- | --- | --- | --- | --- | --- | --- | --- | --- | --- | --- | --- | --- | --- | --- |
| Predictors | OR | 95% CI | | p-value | OR | 95% CI | | p-value | OR | 95% CI | | p-value | OR | 95% CI | | p-value |
| *Health literacy* |  |  |  |  |  |  |  |  |  |  |  |  |  |  |  |  |
| Overall health literacy score ^a^ | **1.04** | **1.02** | **1.06** | **<0.001** | 1.00 | 0.98 | 1.03 | 0.827 | **1.04** | **1.01** | **1.06** | **0.002** | **0.98** | **0.96** | **0.99** | **0.010** |
| *Worry and anxiety* |  |  |  |  |  |  |  |  |  |  |  |  |  |  |  |  |
| Overall worry and anxiety score ^a^ | 0.99 | 0.98 | 1.00 | 0.080 | **0.98** | **0.97** | **1.00** | **0.007** | 1.00 | 0.99 | 1.02 | 0.609 | 1.00 | 0.99 | 1.01 | 0.486 |
| Cognitive symptoms ^a^ |  |  |  |  |  |  |  |  |  |  |  |  |  |  |  |  |
| Yes (Ref. No) | 0.92 | 0.61 | 1.38 | 0.671 | 0.90 | 0.52 | 1.56 | 0.717 | 0.87 | 0.49 | 1.52 | 0.619 | 0.78 | 0.53 | 1.15 | 0.209 |
| Somatic symptoms ^a^ |  |  |  |  |  |  |  |  |  |  |  |  |  |  |  |  |
| Yes (Ref. No) | 0.81 | 0.61 | 1.07 | 0.145 | **0.63** | **0.44** | **0.91** | **0.013** | 1.06 | 0.71 | 1.59 | 0.771 | 0.97 | 0.75 | 1.27 | 0.849 |
| GAD criteria ^a^ |  |  |  |  |  |  |  |  |  |  |  |  |  |  |  |  |
| Yes (Ref. No) | 0.87 | 0.49 | 1.53 | 0.629 | 0.55 | 0.28 | 1.06 | 0.073 | 0.98 | 0.43 | 2.20 | 0.954 | 0.73 | 0.43 | 1.25 | 0.256 |
| No WAQ criteria ^a^ |  |  |  |  |  |  |  |  |  |  |  |  |  |  |  |  |
| Yes (Ref. No) | 1.16 | 0.88 | 1.52 | 0.302 | **1.50** | **1.04** | **2.15** | **0.029** | 1.05 | 0.71 | 1.55 | 0.805 | 1.01 | 0.78 | 1.31 | 0.930 |
| *Personal COVID-19 situation* |  |  |  |  |  |  |  |  |  |  |  |  |  |  |  |  |
| Risk COVID-19 |  |  |  |  |  |  |  |  |  |  |  |  |  |  |  |  |
| Yes (Ref. No) | **1.61** | **1.17** | **2.23** | **0.004** | 1.53 | 0.98 | 2.40 | 0.064 | 0.75 | 0.51 | 1.09 | 0.135 | 1.30 | 0.98 | 1.72 | 0.069 |
| Physical distancing ^b^ |  |  |  | **<0.001** |  |  |  | **0.019** |  |  |  | 0.573 |  |  |  | 0.894 |
| (Self-)isolation | 0.84 | 0.61 | 1.16 |  | 0.88 | 0.58 | 1.34 |  | 0.98 | 0.65 | 1.50 |  | 1.07 | 0.80 | 1.43 |  |
| Physical distancing (Ref.) | **1** |  |  |  | **1** |  |  |  | 1 |  |  |  | 1 |  |  |  |
| No physical distancing | **0.40** | **0.31** | **0.52** |  | **0.61** | **0.43** | **0.86** |  | 0.83 | 0.58 | 1.18 |  | 1.01 | 0.78 | 1.31 |  |
| Contact with COVID-19 positive case ^b^ |  |  |  | 0.760 |  |  |  | **0.038** |  |  |  | **0.020** |  |  |  | 0.435 |
| No (Ref.) | 1 |  |  |  | **1** |  |  |  | **1** |  |  |  | 1 |  |  |  |
| Yes (confirmed) | 0.89 | 0.63 | 1.27 |  | **0.63** | **0.41** | **0.97** |  | 1.19 | 0.73 | 1.96 |  | 1.24 | 0.89 | 1.73 |  |
| Yes (probable) | 1.07 | 0.71 | 1.62 |  | 0.65 | 0.40 | 1.06 |  | **3.24** | **1.40** | **7.48** |  | 1.09 | 0.74 | 1.59 |  |
| Thinking to have had COVID-19 ^a, b^ |  |  |  |  |  |  |  |  |  |  |  |  |  |  |  |  |
| Yes (Ref. No) | 0.83 | 0.58 | 1.17 | 0.279 | **0.49** | **0.33** | **0.73** | **0.001** | 1.62 | 0.93 | 2.82 | 0.086 | 1.10 | 0.79 | 1.53 | 0.587 |
| Pandemic stage at study ^b^ |  |  |  | **<0.001** |  |  |  | 0.135 |  |  |  | 0.211 |  |  |  | 0.926 |
| Extreme restrictions  (up to 10.05.2020; Ref) | **1** |  |  |  | 1 |  |  |  | 1 |  |  |  | 1 |  |  |  |
| Mild easing of restrictions  (11.05.2020-5.06.2020) | 0.84 | 0.66 | 1.06 |  | 0.80 | 0.58 | 1.10 |  | 1.28 | 0.94 | 1.73 |  | 0.96 | 0.77 | 1.19 |  |
| Extensive easing of restrictions  (from 06.06.2020) | **0.36** | **0.22** | **0.58** |  | 1.54 | 0.68 | 3.50 |  | 1.54 | 0.74 | 3.21 |  | 0.98 | 0.61 | 1.57 |  |

Note: Continued on next page.

|  | Daily information-seeking | | | | Use of traditional media | | | | Use of online resources | | | | Use of personal networks | | | |
| --- | --- | --- | --- | --- | --- | --- | --- | --- | --- | --- | --- | --- | --- | --- | --- | --- |
| Predictors | OR | 95% CI | | p-value | OR | 95% CI | | p-value | OR | 95% CI | | p-value | OR | 95% CI | | p-value |
| *Sociodemographic information* |  |  |  |  |  |  |  |  |  |  |  |  |  |  |  |  |
| Sex |  |  |  |  |  |  |  |  |  |  |  |  |  |  |  |  |
| Male (Ref. Female) | **1.67** | **1.28** | **2.18** | **<0.001** | 0.79 | 0.57 | 1.09 | 0.150 | 0.77 | 0.56 | 1.07 | 0.115 | 0.82 | 0.65 | 1.03 | 0.092 |
| Age ^b^ |  |  |  | **<0.001** |  |  |  | **<0.001** |  |  |  | **<0.001** |  |  |  | **0.002** |
| 18-25 years (Ref.) | **1** |  |  |  | **1** |  |  |  | **1** |  |  |  | **1** |  |  |  |
| 26-40 years | 1.31 | 0.88 | 1.95 |  | 1.13 | 0.70 | 1.82 |  | 1.28 | 0.72 | 2.25 |  | **0.57** | **0.38** | **0.86** |  |
| 41-65 years | **2.81** | **1.87** | **4.22** |  | **2.95** | **1.77** | **4.93** |  | 1.26 | 0.72 | 2.23 |  | **0.52** | **0.35** | **0.79** |  |
| 66-90 years | **11.46** | **5.11** | **25.70** |  | **15.48** | **3.58** | **67.04** |  | **0.27** | **0.14** | **0.52** |  | 0.94 | 0.55 | 1.61 |  |
| Highest educational achievement ^a, b^ |  |  |  | 0.593 |  |  |  | 0.259 |  |  |  | **<0.001** |  |  |  | 0.954 |
| Compulsory school | 1.25 | 0.39 | 3.96 |  | 0.71 | 0.20 | 2.54 |  | **0.16** | **0.06** | **0.46** |  | 0.90 | 0.32 | 2.51 |  |
| Vocational training | 0.81 | 0.61 | 1.07 |  | 0.98 | 0.67 | 1.41 |  | **0.32** | **0.23** | **0.46** |  | 1.06 | 0.81 | 1.39 |  |
| Upper secondary education | 1.02 | 0.75 | 1.37 |  | 1.52 | 0.98 | 2.37 |  | **0.52** | **0.35** | **0.76** |  | 1.11 | 0.84 | 1.47 |  |
| University education (Ref.) | 1 |  |  |  | 1 |  |  |  | **1** |  |  |  | 1 |  |  |  |
| Employment ^a, b^ |  |  |  | **<0.001** |  |  |  | **0.016** |  |  |  | **<0.001** |  |  |  | 0.060 |
| Employed (Ref.) | **1** |  |  |  | **1** |  |  |  | **1** |  |  |  | 1 |  |  |  |
| Unemployed | **2.36** | **1.52** | **3.66** |  | **2.50** | **1.29** | **4.84** |  | **0.28** | **0.19** | **0.41** |  | 1.22 | 0.87 | 1.71 |  |
| In education | **0.57** | **0.35** | **0.91** |  | 0.79 | 0.43 | 1.47 |  | 0.66 | 0.35 | 1.26 |  | 1.72 | 1.05 | 2.80 |  |
| *Interactions with health literacy* |  |  |  |  |  |  |  |  |  |  |  |  |  |  |  |  |
| Employment * Health literacy ^b^ |  |  |  |  |  |  |  | **0.031** |  |  |  |  |  |  |  |  |
| Employed (Ref.) |  |  |  |  | **1** |  |  |  |  |  |  |  |  |  |  |  |
| Unemployed |  |  |  |  | **1.18** | **1.04** | **1.33** |  |  |  |  |  |  |  |  |  |
| In education |  |  |  |  | 1.03 | 0.95 | 1.12 |  |  |  |  |  |  |  |  |  |
| Thinking to have already had COVID-19 * Health literacy |  |  |  |  |  |  |  |  |  |  |  |  |  |  |  |  |
| Yes (Ref. No) |  |  |  |  |  |  |  |  |  |  |  |  | **1.11** | **1.05** | **1.18** | **<0.001** |

Abbreviations: CI, confidence interval; GAD, generalized anxiety disorder; OR, odds ratio; WAQ, worry and anxiety questionnaire.

^a^ Variables that had missing data: Overall health literacy score (n=8); Thinking to have already had COVID-19 (n=5); Educational achievement (n=4); Employment (n=2); Overall worry and anxiety score (n=416); Cognitive symptoms (n=410); Somatic symptoms (n=416); GAD criteria (n=425); No WAQ criteria (n=425).

^b^ Global p-value from Wald test.

Note: The number of participants included in each univariable model varies because of missing values. Statistically significant variables (at level α<0.05) are highlighted in bold. For space reasons, only significant interactions with health literacy were reported in this table.
